# Lipids in HIV's Envelope Help the Virus to Spread

**DOI:** 10.1371/journal.pbio.1001316

**Published:** 2012-04-24

**Authors:** Caitlin Sedwick

**Affiliations:** Freelance Science Writer, San Diego, California, United States of America

## Abstract

An accessible sialyllactose moiety on viral membrane gangliosides is shown to be essential for HIV-1 uptake into mature dendritic cells, thereby promoting viral transfer and infection of bystander CD4+ T lymphocytes.

**Figure pbio-1001316-g001:**
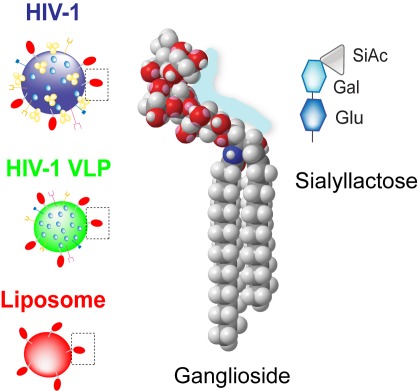
**Left: Gangliosides in HIV's lipid envelope (red) promote uptake of the virus (or experimental substitutes) by dendritic cells.** **Middle: a space-filling model of a ganglioside. Right: a schematic of a simple ganglioside's head group. Image credit: F.-Xabier Contreras, Maier Lorizate, and Nuria Izquierdo-Useros.**

[Fig pbio-1001316-g001]Dendritic cells (DCs) are a type of immune cell that patrol tissues, on the lookout for microbial invaders. When DCs encounter a pathogen, they chop it up into tiny pieces and then carry samples of it to local lymph nodes. There, they display their finds to another kind of immune cell, the T cell, which then mounts a full-fledged immune response against the invader.

Unfortunately, HIV, the virus that causes AIDS, can exploit the DC surveillance network to its own advantage: HIV is picked up by DCs that patrol mucosal tissues, but avoids being killed by them, and instead hitches a ride to lymph nodes. There, the virus transfers into its favorite host cell type, the T cell. Until now, it wasn't clear how DCs recognize HIV for uptake—a mystery that's now been solved thanks to a joint effort by Spanish and German research groups. The groups, headed by Nuria Izquierdo-Useros and Javier Martinez-Picado in Spain, and Maier Lorizate and Hans-Georg Kräusslich in Germany, uncover the culprit in this month's issue of *PLoS Biology*.

It's no mystery how DCs detect many pathogens: the microbes betray themselves by displaying certain pathogen-specific compounds on their surfaces, which DCs recognize using dedicated receptors. HIV also displays a few viral proteins on its surface (it hides the rest away under a lipid bilayer envelope that it acquires from the plasma membrane of infected cells). However, previous studies indicated that viral proteins displayed on the surface may not be essential for DC capture. Instead, prior work from Martinez-Picado's group led the authors to theorize that host cell glycosphingolipids incorporated into the virus envelope could be critical in triggering DC recognition.

There are many types of glycosphingolipids, and one kind had previously been observed in the membranes of several retroviruses, including HIV. This lipid, GM3, is from a subclass of glycosphingolipids called gangliosides. To follow up on these findings, Izquierdo-Useros, Lorizate, and colleagues first examined whether other gangliosides might also be present in HIV's envelope. Indeed, they found several other gangliosides there—an observation that prompted the researchers to examine what role gangliosides might play in HIV recognition and capture by DCs.

To determine whether gangliosides are needed for HIV uptake by DCs, the authors examined DC uptake of artificial virus-like particles (VLPs), which carry a lipid envelope and the inner structural proteins, but lack HIV surface glycoproteins and viral genetic material. They found that VLPs containing gangliosides were able to get into DCs, but those lacking gangliosides could not do so. Next, the researchers tested which gangliosides can promote DC uptake by using artificial lipid globules called liposomes, each containing a different ganglioside. Similar to VLPs, DCs easily took up liposomes containing gangliosides, but were unable to take up liposomes devoid of all gangliosides. Both liposomes and VLPs, the authors showed, use the same method to enter DCs—a method that depends not on viral proteins but on the presence of gangliosides in the viral envelope.

These data suggest that there is some special feature of gangliosides that promotes their specific recognition by DCs. While much of a ganglioside is buried in the lipid bilayer, a portion of it, called the head group, is exposed at the membrane surface. Ganglioside head groups contain one or more copies of sialic acid, a sugar, with different ganglioside types each sporting distinct arrangements of their sialic acids. The authors' investigation revealed that it's these head groups—particularly sialic acids attached to a lactose group—that promote DC recognition. But, while DC uptake requires sialic acid molecules, gangliosides whose head groups are too complex can't be recognized by DCs.

These findings all point to the idea that gangliosides are essential for HIV uptake by DCs. In fact, the authors showed that addition of free GM3 head groups to cell cultures prevented DCs from picking up intact HIV. They also demonstrated that DCs exposed to ganglioside-depleted HIV can't pick up the virus and so can't efficiently transfer it to T cells. Therefore, gangliosides may also affect HIV's ability to establish infection in the body. Of course, further studies are needed to understand how DCs recognize gangliosides in the viral envelope, how HIV avoids destruction after ganglioside-mediated DC uptake, and how the virus later escapes to infect T cells. Nonetheless, gangliosides may represent a useful therapeutic target for preventing or limiting infection by HIV and other ganglioside-containing viruses.

**Izquierdo-Useros N, Lorizate M, Contreras F-X, Rodriguez-Plata MT, Glass B, et al. (2012) Sialyllactose in Viral Membrane Gangliosides Is a Novel Molecular Recognition Pattern for Mature Dendritic Cell Capture of HIV-1. doi:10.1371/journal.pbio.1001315**

